# Assessment for apraxia in mild cognitive impairment and Alzheimer's dise

**DOI:** 10.1590/S1980-57642015DN91000011

**Published:** 2015

**Authors:** Mirela Ward, Juliana F. Cecato, Ivan Aprahamian, José Eduardo Martinelli

**Affiliations:** 1 Geriatrics Section, Department of Internal Medicine, Faculty of Medicine of Jundiaí, Jundiaí, State of São Paulo, Brazil.

**Keywords:** apraxia, neuropsychometric tests, elder, Alzheimer's disease, mild cognitive impairment, diagnosis

## Abstract

**Objective:**

To evaluate apraxia in healthy elderly and in patients diagnosed with Alzheimer's disease (AD) and Mild cognitive impairment (MCI).

**Methods:**

We evaluated 136 subjects with an average age of 75.74 years (minimum 60 years old, maximum 92 years old) and average schooling of 9 years (minimum of 7 and a maximum of 12 years), using the Mini-Mental State examination (MMSE), Cambridge Cognitive Examination (CAMCOG) and the Clock Drawing Test. For the analysis of the presence of apraxia, eight subitems from the CAMCOG were selected: the drawings of the pentagon, spiral, house, clock; and the tasks of putting a piece of paper in an envelope; the correct one hand waiving "Goodbye" movements; paper cutting using scissors; and brushing teeth.

**Results:**

Elder controls had an average score of 11.51, compared to MCI (11.13), and AD patients, whose average apraxia test scores were the lowest (10.23). Apraxia scores proved able to differentiate the three groups studied (p=0.001). In addition, a negative correlation was observed between apraxia and MMSE scores.

**Conclusion:**

We conclude that testing for the presence of apraxia is important in the evaluation of patients with cognitive impairments and may help to differentiate elderly controls, MCI and AD.

## Abstract (pt)

**Objetivo:**

Avaliar apraxia em idosos saudáveis, com diagnóstico de doença de Alzheimer (DA) e Comprometimento Cognitivo Leve (CCL).

**Métodos:**

Foram avaliados 136 indivíduos com uma idade média de 75,74 anos (mínimo de 60 anos de idade, máximo 92 anos) e escolaridade média de 9 anos (mínimo de 7 e máximo de 12 anos), por meio do Mini-exame do Estado Mental (MEEM), Cambridge Cognitive Examination (CAMCOG) e o teste do relógio. Para analisar a presença de apraxia, foram selecionados oito subitens do CAMCOG: os desenhos do Pentágono, da espiral, da casa, do relógio, e também a tarefa de colocar um pedaço de papel em um envelope e os movimentos corretos com uma mão para dar "adeus", cortar papel com uma tesoura e escovar os dentes.

**Resultados:**

Idosos saudáveis sem alterações cognitivas apresentaram média de 11,51, em comparação com CCL (11,13), e DA o qual apresentou pior média no teste de apraxia (10.23). O subteste de apraxia diferenciou os três grupos diagnósticos (p=0,001). Observou-se uma correlação negativa entre os escores de apraxia e os do MEEM.

**Conclusão:**

Conclui-se que a investigação da presença de apraxia é importante na avaliação cognitiva de pacientes com comprometimento cognitivo e pode ser útil em diferenciar controles idosos, indivíduos com CCL e com DA.

## INTRODUCTION

According to the National Institute of Neurological and Communicative Disorders and Stroke and the Alzheimer's Disease and Related Disorders Association (NINCDS-ADRDA), the diagnosis of Alzheimer's disease (AD) relies on deficits in two or more areas of cognition with progressive worsening of memory and other cognitive functions. The diagnosis of probable AD is supported by progressive deterioration of other specific cognitive functions such as language (aphasia), motor skills (apraxia) and perception (agnosia).^1^ Hence, testing for these cognitive functions, including apraxia, is a crucial part of the dementia diagnostic assessment.^2^

Apraxia has a wide spectrum of disorders with the common inability to perform a skilled or learned act and several types have been described, including a limb-kinetic type, which is a form of loss of hand and finger dexterity resulting from inability to connect or isolate individual movements.^3-6^ Ideomotor apraxia is characterized by the inability to correctly imitate hand gestures and voluntarily use tools. Ideational/conceptual apraxia is a form in which there is an inability to perform a series of acts in the due sequence (for instance, to insert a sheet of paper into an envelope) or an inability to appropriately use a tool (for instance to use a pair of scissors).^7,8^ Another type of apraxia is constructional apraxia, in which there is difficulty drawing simple figures or assembling blocks to form a design. The term visuoconstructive disability is frequently used and encompasses constructional apraxia. Recognizing one type of apraxia does not exclude other concurrent types, and multiple kinds of apraxia can be diagnosed in the same patient.^4-6^

Psychomotor activity impairment and motor function difficulties generated by apraxia are some of the most distressful features of AD.^3,7-9^ This neurological deficit causes a loss of ability to perform precise movements and gestures, hence impeding the patient to accomplish a learned purposeful complex act correctly.^7-9^ In general, apraxia advances in step with dementia and examiners should always remember to assess the patient's ability, for example, to write sentences, draw, fold a sheet of paper, drink water, move the upper and lower limbs, and other tasks of progressive difficulty in order to characterize dementia stage.^7-11^

However, there is no accurate instrument for measuring apraxia in aging patients. In fact, most elder individuals tend to display movement and speech impairments both because of dementia and due to a physiologic deterioration of muscular and central nervous system functions.^10^ In addition, apraxia features are closely related to the patient's educational level and activity.^11^ Also, the diagnosis of apraxia is difficult to characterize in patients with mild cognitive impairment (MCI), defined by the American Academy of Neurology as the presence of memory complaints and memory impairment in individuals still presenting normal global cognitive functioning and intact activities of daily living.^1^

On the other hand, apraxia assessment may help define the severity of the dementia and predict its progression.^12^ In addition, the presence of apraxia is important for planning stimulatory therapies such as physiotherapy or occupational therapy.

The aim of this study was to verify the presence of apraxia in MCI and AD patients and its relationship to performance on other cognitive tests and impact on activities of daily living.

## METHODS

This cross-sectional study was conducted in the Department of Geriatrics and Gerontology at the Medical School of Jundiai from January 2011 to January 2014 and included 136 consecutive individuals aged 60 years or more with at least four years of schooling who sought medical care and agreed to participate by signing an informed consent form. Patients were classified as probable and possible, or only probable, AD when they met the NINCDS-ADRDA criteria, and as MCI according to the criteria of Petersen.^21^ Patients with severe dementia (Clinical Dementia Rating=3), history of stroke, Parkinson disease features, hand palsies, visual and auditory impairments or depression were excluded. A control group (CG) of healthy elderly was formed comprising individuals whose performance on the neuropsychological tests exceeded the respective cut-off points and who did not present depressive symptoms or impairments in daily activities.

Both the AD and MCI groups of patients as well as the controls were submitted to a detailed in-person clinical anamnesis; neuroimaging; laboratory; and neuropsychiatric evaluation including the Cambridge Cognitive Examination (CAMCOG);^13^ the Mini-Mental State Examination (MMSE);^14,15^ the Clock Drawing Test (CDT) ranked according to both the Mendez^16^ and Shulman^17^ scales; and the Geriatric Depression Scale.^18^ The performance of daily activities was assessed by the Pfeffer Functional Activities Questionnaire (PFAQ).^13,19^

Eight CAMCOG test sub-items were selected for the evaluation of apraxia. These items were: the drawings of the pentagon, spiral, house, clock; and the tasks of: inserting a sheet of paper into an envelope; the correct one hand movements designed to wave "goodbye"; cutting a sheet of paper with a pair of scissors; and brushing teeth. Scores attributed to each one of these sub-items are described in Table 1. Low total scores, revealing bad performance, were considered indicative of apraxia.

**Table 1:** Sub-items of CAMCOG test employed for apraxia evaluation.

Apraxia	Score
Drawing of the pentagon	1 point
Drawing of the spiral	1 point
Drawing the house	1 point
Drawing a clock	3 points
Putting a sheet of paper in an envelope	3 points
“Goodbye” – correct movement	1 point
Scissors – correct movement	1 point
Brushing teeth – correct movement	1 point
Total	12 points

**Statistical analyses.** The data obtained were analyzed with the SPSS (15.0) program. Normality was assessed using the Kolmogorov-Smirnov test and was observed for all measures in each one of the groups investigated. Age among groups was compared using the Kruskal-Wallis test whereas education level and gender was assessed using the Chi-square test. Student-Newman-Keuls post-hoc analysis was performed to differentiate the diagnostic groups. Significance level was set at 5% (p). Comparative analyses of the three patient groups was also performed using Pearson's correlation coefficient (r) for age and cognitive tests (MMSE, CAMCOG and CDT).

## RESULTS

Mean age of the participants was 75.7±7.38 years, most elders were female (65.4 %), and the groups did not differ for age and gender distribution. Individuals in the control group had a greater number of years of schooling (Table 2).

**Table 2:** Demographic and schooling features of the 52 patients diagnosed with Alzheimer’s disease (AD); the 45 patients considered as having mild cognitive impairment (MCI) and the 39 healthy control individuals (CG).

		MCI	AD	CG	p
Age years (range)		76.60±7.06 (63-92)	77.92±6.97 (64-91)	71.82±6.89 (60-89)	0.185*
Gender (%)	Female	27 (60)	33 (63.5%)	29 (74.4%)	0.358 **
	Male	18 (40)	19 (36.5%)	10 (25.6%)	
Schooling	5 to 8 years	23 (51.1%)	21 (40.4%)	12 (30.8%)	0.040**
	> 9 years	22 (48.9%)	31 (59.6%)	27 (69.2%)	

* p: Kruskal-Wallis;

** p: Chi-square test.

The neurological assessment using the CAMCOG, MMSE and apraxia results, summarized in Table 3, demonstrated that patients in the AD group had lower scores (indicating more severe impairment) compared to the MCI and to the healthy control groups of elders. In fact, apraxia scores were able to distinguish the three diagnostic groups (p<0.0001). Patients in the AD group had scores below the cut-off point for the CAMCOG (> 80 points). Also, MCI patients' CAMCOG scores were higher than expected.

**Table 3:** Description of MMSE, CAMCOG and apraxia assessment of the 52 patients diagnosed with Alzheimer Disease (AD); the 45 patients considered as having a mild cognitive impairment (MCI) and the 39 healthy control individuals (CG).

Test	CG Mean±SD (Min-Max)	MCI Mean±SD (Min-Max)	AD Mean±SD (Min-Max)	p
MMSE	29.10±1.16 (26-30)	26.93±2.07 (21-30)	23.40±3.87 (14-29)	0.0001
CAMCOG	97.74±5.36 (82-107)	88.40±6.63 (73-100)	77.2±11.53 (52-96)	0.0001
Apraxia	11.51±0.72 (10-12)	11.13±1.08 (8-12)	10.23±1.98 (2-12)	0.001

p: Kruskal-Wallis test; Min: minimum; Max: maximum; SD: Standard deviation. MMSE: Mini mental State Examination.

Age (p=0.185; Kruskal-Wallis) and gender (p=0.358; Chi-square test) did not influence apraxia assessment in the three diagnostic groups. Only schooling years influenced the assessment significantly (p=0.040; Chi-square test). There was a significant, albeit moderate correlation between the apraxia tests and the MMSE (r=0.40, p<0.0001) and CAMCOG (r=0.45, p<0.0001), CDT-Mendez (r=0.50, p<0.0001) and CDT-Shulman (r=0.54, p<0.0001), as shown in Table 4.

**Table 4:** Correlations between cognitive tests and apraxia with and without adjustment for schooling years in individuals from the Control group.

		Age	PFAQ	MMSE	CAMCOG	Mendez	Shulman
Apraxia	Pearson correlation	–0.145	–0.182	0.312	0.627	0.310	0.216
	Sig. (2-tailed)	0.378	0.268	0.053	0.000	0.055	0.187
	N	39	39	39	39	39	39
Apraxia controlled by schooling	Correlation	–0.172	–0.108	0.234	0.578	0.207	0.097
	Significance (2-tailed)	0.301	0.517	0.157	0.000	0.212	0.561
	Df	36	36	36	36	36	36

r: Pearson correlation coefficient; p: χ^2^; MMSE: Mini-mental State Examination; PFAQ: Pfeffer Functional Activities Questionnaire; Mendez: CDT Mendez scoring scale; Shulman: CDT Shulman scoring scale.

The results on the Apraxia assessment, depicted in Table 4, were associated with CAMCOG results in the control group. This association remained significant when apraxia was controlled by schooling and age. Concerning the MCI group, depicted in Table 5, apraxia was associated with the performance on the CAMCOG and Shulman-CDT tests where both associations remained significant even after controlling for the number of years of schooling. In the AD group, depicted in Table 6, there was a significant association between apraxia and both Mendez-CDT and Shulman-CDT results. These data suggest that the worse the clinical dementia, the more apraxia problems appear, tending to impair even straight-forward tasks such as drawing.

**Table 5:** Correlations between cognitive tests and apraxia with and without adjustment for schooling years in individuals from the Mild Cognitive Impairment (MCI) group.

		Age	PFAQ	MMSE	CAMCOG	Mendez	Shulman
Apraxia	Pearson correlation	–0.193	–0.001	0.191	0.472	0.160	0.377
	Sig. (2-tailed)	0.204	0.997	0.208	0.001	0.293	0.011
	N	45	45	45	45	45	45
Apraxia controlled by schooling	Correlation	–0.321	0.010	0.165	0.466	0.083	0.307
	Significance (2-tailed)	0.034	0.947	0.285	0.001	0.594	0.043
	Df	42	42	42	42	42	42

r: Pearson correlation coefficient; p: χ^2^; MMSE: Mini-mental State Examination; PFAQ: Pfeffer Functional Activities Questionnaire; Mendez: CDT Mendez scoring scale; Shulman: CDT Shulman scoring scale.

**Table 6:** Correlations between cognitive tests and apraxia with and without adjustment for schooling year in individuals from the Alzheimer’s disease group.

		Age	PFAQ	MMSE	CAMCOG	Mendez	Shulman
Apraxia	Pearson Correlation	0.135	0.073	0.289*	0.214	0.539	0.537
	Sig. (2-tailed)	0.340	0.605	0.040	0.135	0.000	0.000
	N	52	52	51	50	51	51
Apraxia controlled by schooling	Correlation	0.171	0.043	0.288	0.203	0.525	0.519
	Significance (2-tailed)	0.239	0.770	0.044	0.162	0.000	0.000
	Df	47	47	47	47	47	47

r: Pearson correlation coefficient; p: χ^2^; MMSE: Mini-mental State Examination; PFAQ: Pfeffer Functional Activities Questionnaire; Mendez: CDT Mendez scoring scale; Shulman: CDT Shulman scoring scale.

In the control group, cognitive variability was greater than that of apraxia, which is understandable since the cognitive system is preserved. Once cognition starts to decline, apraxia starts to become a problem; as cognitive variability was reduced because of AD progression, apraxia variability increased and new test relationships were found, e.g. for specific praxis tests such as the CDT. In fact, the CDT proved to be the best test to support apraxia evaluation, even after adjusting statistical comparisons by schooling years.

No effect of apraxia on functional activities, as assessed by the Pfeffer scale, was evident.

## DISCUSSION

We demonstrated that MCI and AD patients performed worse than healthy controls on apraxia assessment tests. This worse performance was independent of factors such as age and schooling.

It is important to point out that constructional apraxia was more prevalent among AD patients^21^ in this study. Additionally, our data corroborates findings of previous studies indicating that other types of apraxia are also worse in patients with AD, especially the ideomotor type.^23,25,27^

In the case of the present study, taking into account that both the MMSE and CAMCOG make greater use of writing and drawing apraxia testing, both types of common apraxia (constructional and ideomotor) may be involved. The failure to differentiate between the healthy elderly and those with AD may have been due to the fact that apraxia is not usually found in the early stages of the disease^28^. Future studies should focus on apraxia type differences among the three groups, since general measures of apraxia showed significant differences.^25-28^

Apraxia evaluation is useful in the diagnosis of dementia, but is not a decisive factor since normal aging involves a gradual decline in cognitive function.^29,30^ As previously mentioned, this decline in cognitive functions is dependent on educational factors, health, personality and specific capacity,^29-31^ explaining the relatively high rate in the control group and the difference between patients with AD and MCI. We demonstrated that apraxia was able to differentiate mild cognitive impairment from dementia cases. In fact, Sá et al.^32^ also found that apraxia tests were able to differentiate cases of dementia, both in the early and late stages of the disease, probably due to the involvement of the posterior hemisphere in early stages of AD.^33^

In conclusion, we demonstrated that apraxia was present in MCI and early phases of AD. Apraxia was best detected in MCI and AD by means of CDT scores and new cut-off points for this aspect in these patients suggests the need for further research. It is also important to assess apraxia to aid planning of rehabilitation.

Apraxia assessment has become an important aspect of neurodegenerative diseases and a major indicator for psychotherapy and occupational therapy, contributing to the quality of life of elderly primarily with cognitive decline. In many cases, apraxia may be one of the early symptoms of AD, as shown in this study, where patients with MCI showed decline on apraxia tests. We conclude that apraxia should be better assessed on cognitive tests in older adults with dementia who may also benefit from therapies, thus reducing impact of the disease on activities of daily living.
